# Supplementary figures and images for: Presence of an ultra-small microbiome in fermented cabbages
Source: PeerJ. 2023 Jul 17;11:e15680. doi: 10.7717/peerj.15680 (PMC10358336; doi:10.7717/peerj.15680)

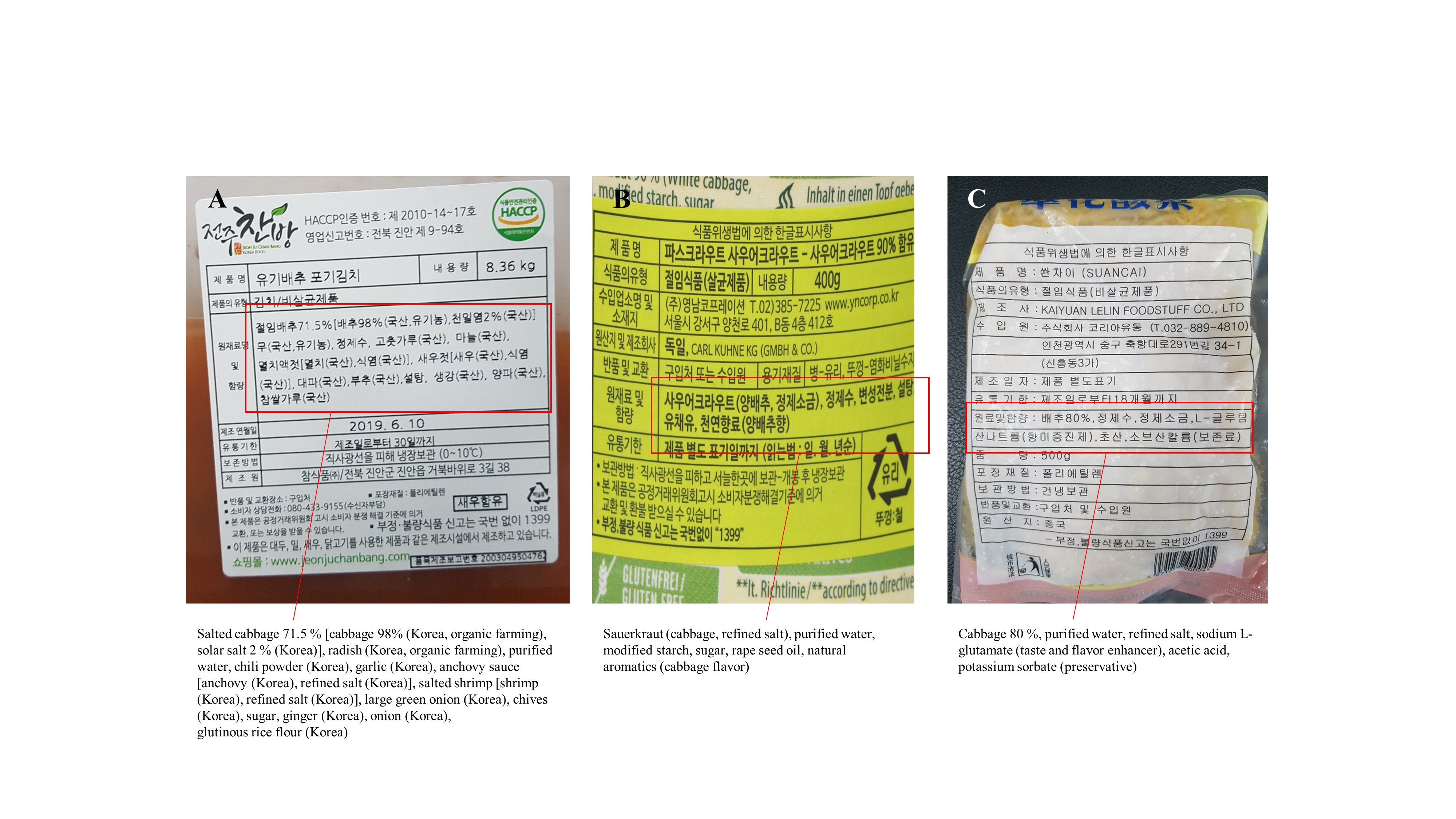

Supplement: Figure S1 — The red boxes represent the ingredients. (A) kimchi, (B) sauerkraut, (C) suancai. [file peerj-11-15680-s001.jpg]

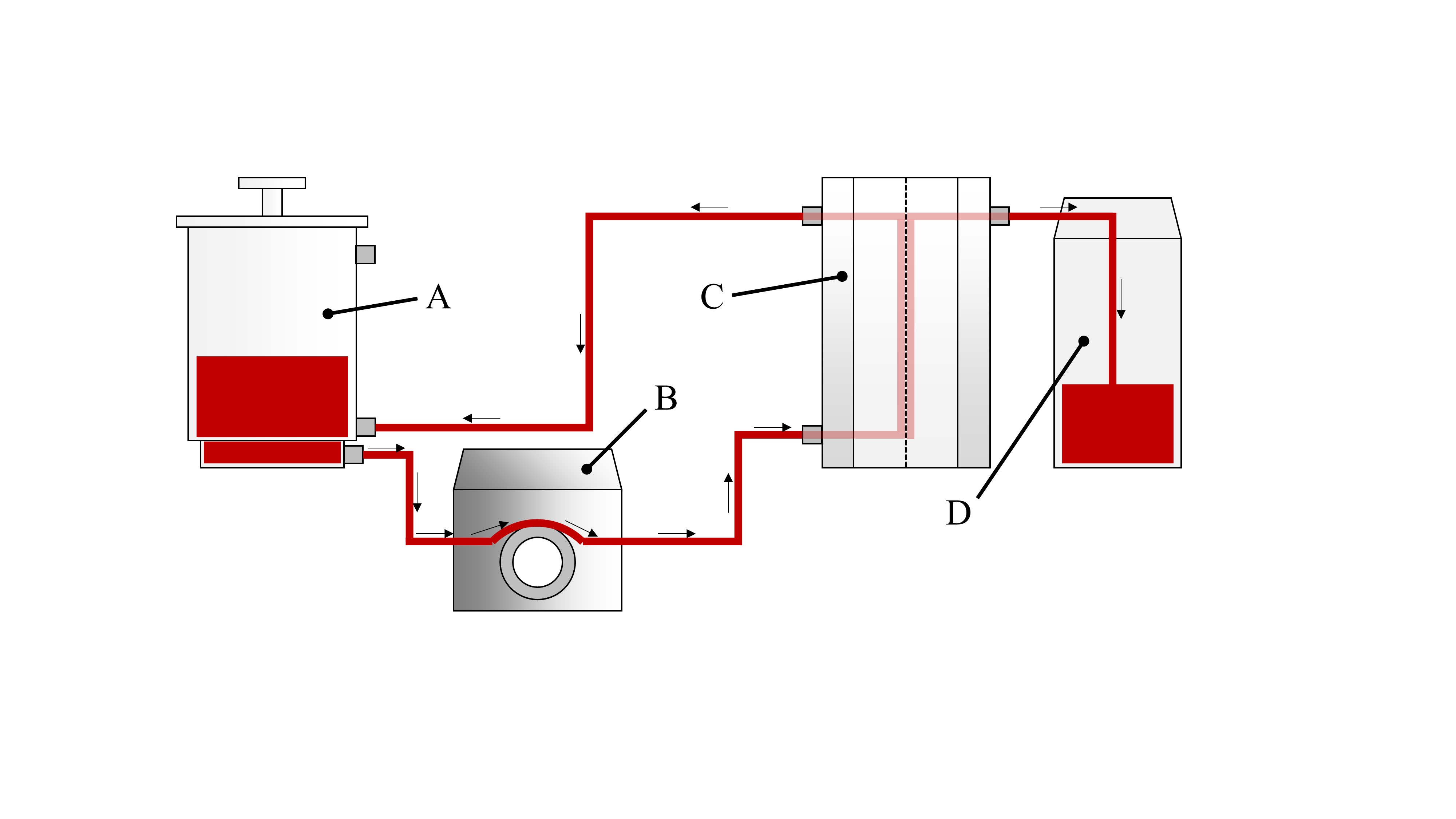

Supplement: Figure S2 — (A) Tank. The solution to be filtered is added. The unfiltered solution is then collected and concentrated via TFF. (B) Peristaltic feed pump. The solution is pumped through the filter membrane by a peristaltic feed pump. (C) TFF cartridge and holder, and where TFF filtration takes place. Filtration was performed with a 0.22 µm pore size or a 100 K molecular weight cut-off (MWCO) filter cartridge installed. (D) Filtrate collection container where the filtered solution is collected. [file peerj-11-15680-s002.jpg]

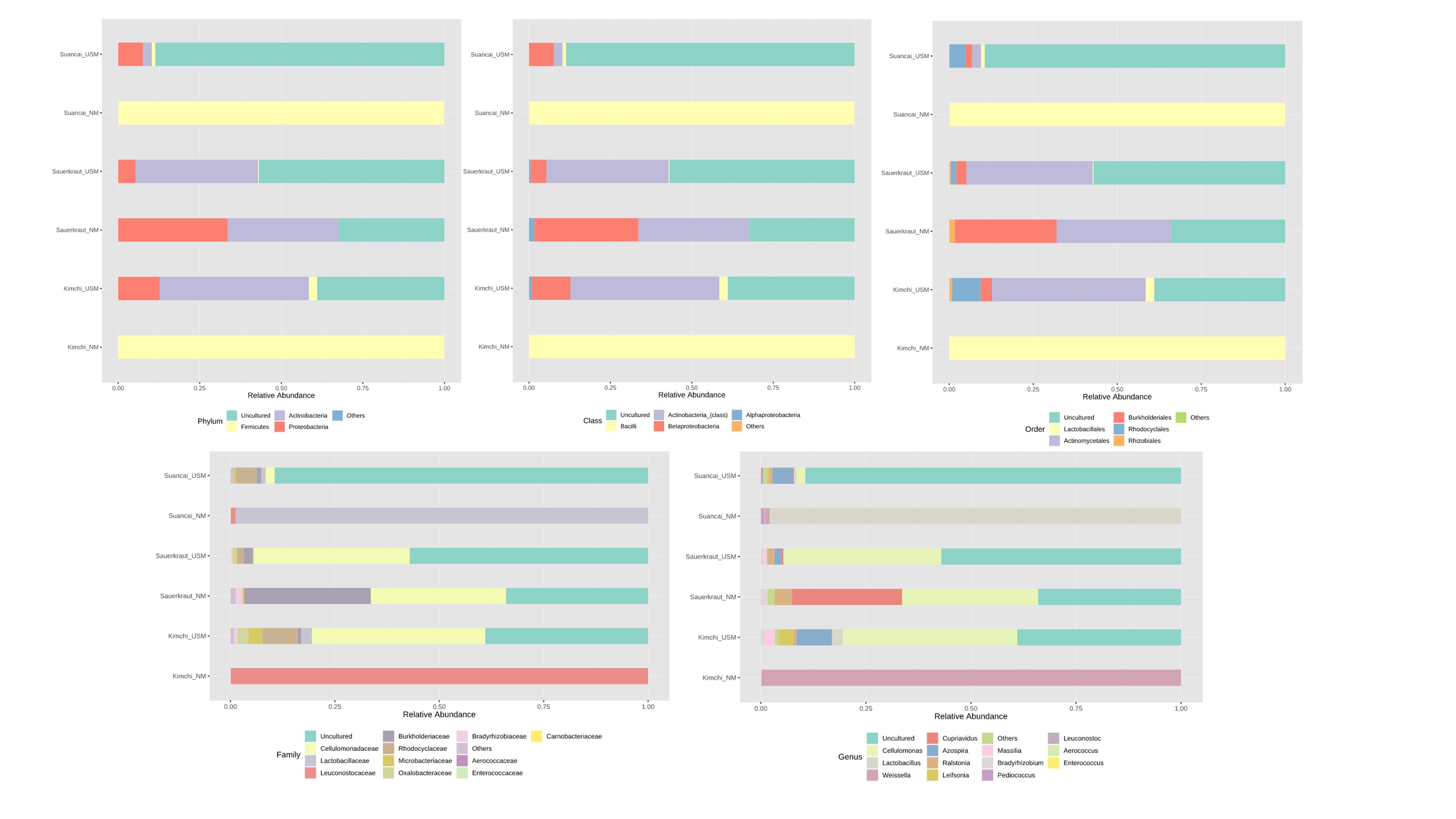

Supplement: Figure S3 [file peerj-11-15680-s003.jpg]

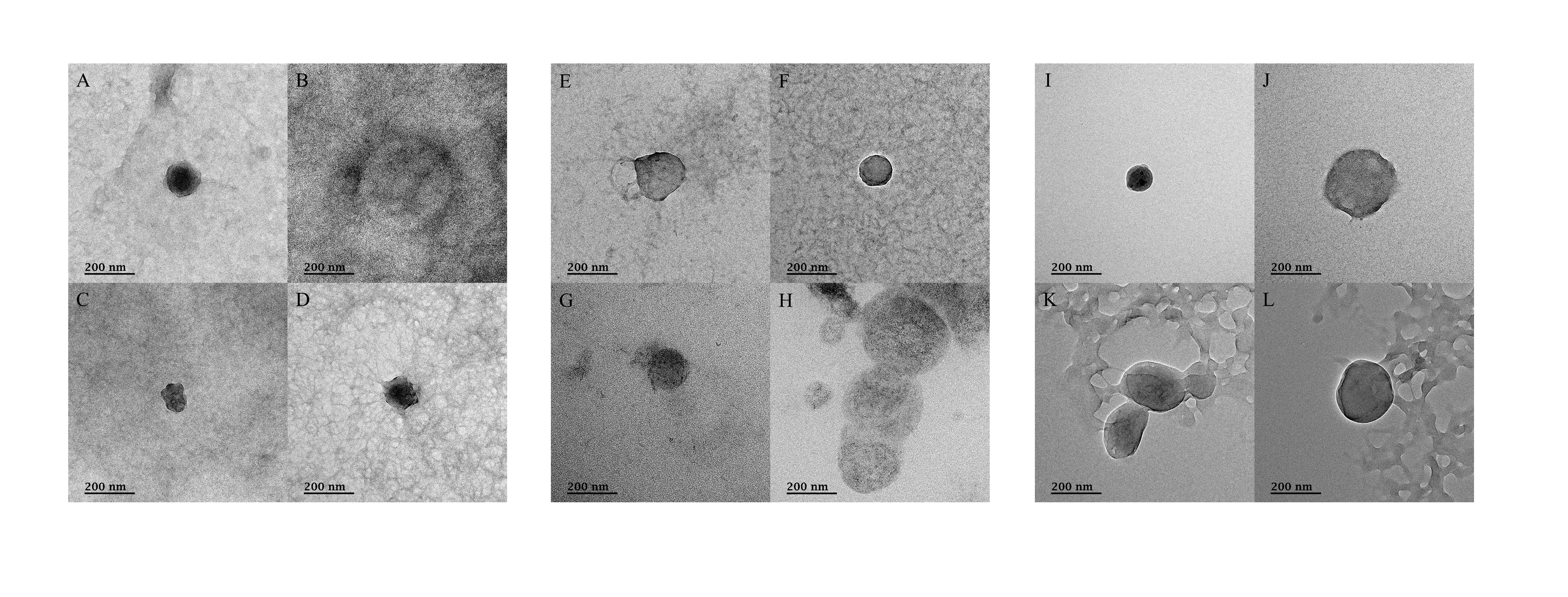

Supplement: Figure S4 — Transmission electron micrographs of the ultramicrobial community of a size < 0.2 µm in (A–D) kimchi (Kimchi_USM), (E–H) sauerkraut (Sauerkraut_USM), and (I–L) suancai (Suancai_USM). [file peerj-11-15680-s004.jpg]
